# Functional and Genetic Characterization of Neuropeptide Y-Like Receptors in *Aedes aegypti*

**DOI:** 10.1371/journal.pntd.0002486

**Published:** 2013-10-10

**Authors:** Jeff Liesch, Lindsay L. Bellani, Leslie B. Vosshall

**Affiliations:** 1 Laboratory of Neurogenetics and Behavior, The Rockefeller University, New York, New York, United States of America; 2 Howard Hughes Medical Institute, The Rockefeller University, New York, New York, United States of America; Johns Hopkins Bloomberg School of Public Health, United States of America

## Abstract

**Background:**

Female *Aedes aegypti* mosquitoes are the principal vector for dengue fever, causing 50–100 million infections per year, transmitted between human and mosquito by blood feeding. *Ae. aegypti* host-seeking behavior is known to be inhibited for three days following a blood meal by a hemolymph-borne humoral factor. Head Peptide-I is a candidate peptide mediating this suppression, but the mechanism by which this peptide alters mosquito behavior and the receptor through which it signals are unknown.

**Methodology/Principal Findings:**

Head Peptide-I shows sequence similarity to short Neuropeptide-F peptides (sNPFs) that have been implicated in feeding behaviors and are known to signal through Neuropeptide Y (NPY)-Like Receptors (NPYLRs). We identified eight NPYLRs in the *Ae. aegypti* genome and screened each in a cell-based calcium imaging assay for sensitivity against a panel of peptides. Four of the *Ae. aegypti* NPYLRs responded to one or more peptide ligands, but only NYPLR1 responded to Head Peptide-I as well as sNPFs. Two NPYLR1 homologues identified in the genome of the Lyme disease vector, *Ixodes scapularis*, were also sensitive to Head Peptide-I. Injection of synthetic Head Peptide-I and sNPF-3 inhibited host-seeking behavior in non-blood-fed female mosquitoes, whereas control injections of buffer or inactive Head Peptide-I [Cys10] had no effect. To ask if NPYLR1 is necessary for blood-feeding-induced host-seeking inhibition, we used zinc-finger nucleases to generate five independent *npylr1* null mutant strains and tested them for behavioral abnormalities. *npylr1* mutants displayed normal behavior in locomotion, egg laying, sugar feeding, blood feeding, host seeking, and inhibition of host seeking after a blood meal.

**Conclusions:**

In this work we deorphanized four *Ae. aegypti* NPYLRs and identified NPYLR1 as a candidate sNPF receptor that is also sensitive to Head Peptide-I. Yet *npylr1* alone is not required for host-seeking inhibition and we conclude that other receptors, additional peptides, or both, regulate this important behavior.

## Author Summary

Female mosquitoes are responsible for spreading many deadly infectious diseases including malaria, dengue fever, and yellow fever. These mosquitoes require a blood meal to produce eggs and preferentially feed on humans, thereby spreading disease as they feed. Females of the dengue vector mosquito *Aedes aegypti* undergo a natural change in behavior after a blood meal in which they lose attraction to humans for over three days. We are interested in understanding this natural behavioral inhibition because it may provide an opportunity to control mosquito blood-feeding behavior. Previous work showed that a small protein called Head Peptide-I could mimic this behavioral inhibition when injected into non-blood-fed females, which normally show very high attraction to humans. In this work, we set out to find the Head Peptide-I receptor and ask if it causes this behavioral inhibition. By testing eight different candidate receptors, we found one called NPYLR1 that responds to Head-Peptide I but is much more sensitive to another peptide called sNPF-3. We made mutant mosquitoes that lack the *npylr1* gene and found that the mutants showed normal sugar- and blood-feeding behavior. We conclude that there must be additional receptors and/or peptides that together cause this long-lasting inhibition of female mosquito attraction to humans.

## Introduction

*Aedes aegypti* mosquitoes are the principal vector for dengue fever, causing 50–100 million infections among humans per year [1]. Female mosquitoes are efficient transmitters of disease because they feed on human blood to obtain necessary nutrients for egg development and can feed on multiple hosts in their lifetime. To locate a host, mosquitoes use a multi-sensory approach that includes detecting visual, olfactory, thermosensory, and gustatory cues to guide a series of behaviors collectively known as host-seeking behavior [2], [3].

**Figure 1:**
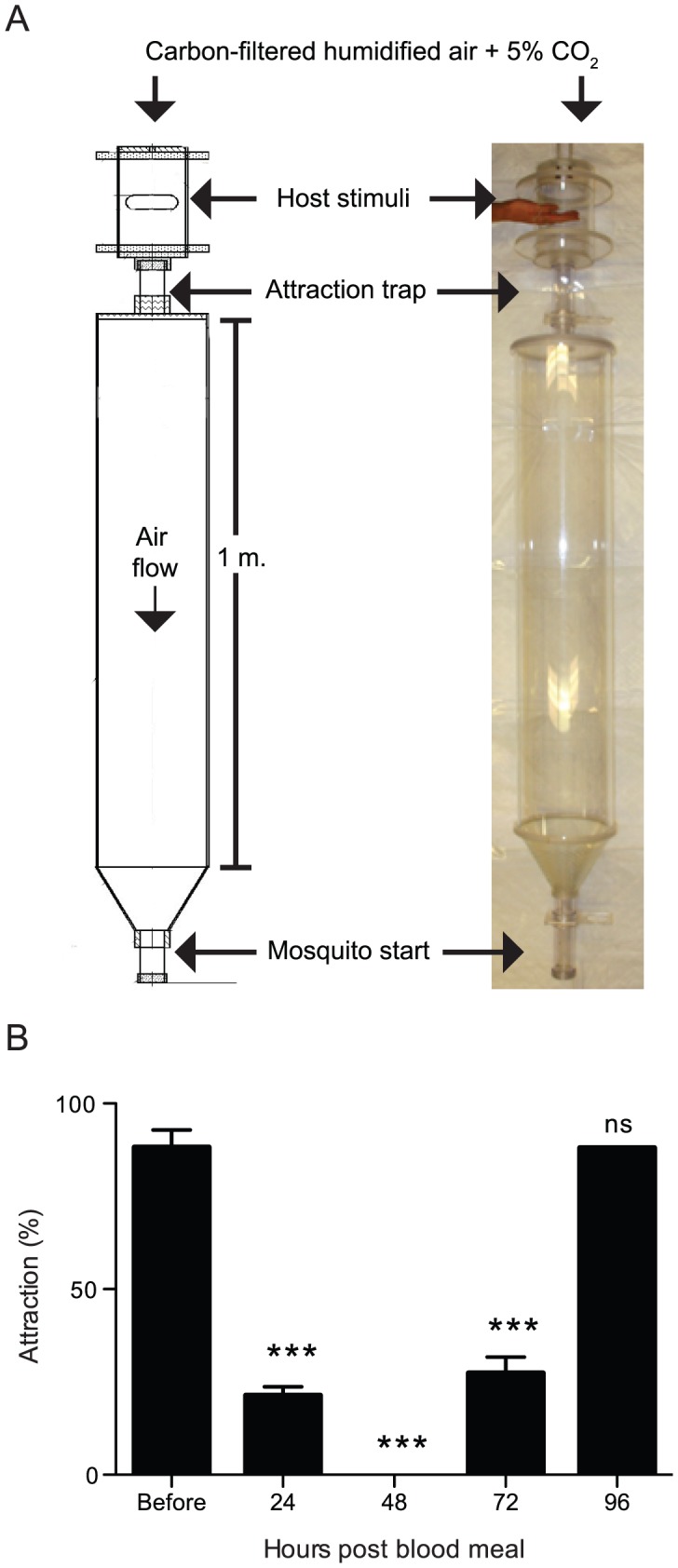
Natural mechanisms for female host-seeking inhibition after a blood meal. (A) Diagram (left) and photograph (right) of the uniport olfactometer with a human hand presented as stimulus. (B) Attraction of female *Ae. aegypti* to a human host throughout the gonotrophic cycle (n = 3–5; ∼20 females per trial). Access to egg laying locations was permitted after 72 hours and observed at 96 hours. Data are plotted as mean±standard error of the mean (SEM). ANOVA with Dunnett's Correction for Multiple Comparison; *** = p<0.001, ns = not significant.

Behavioral studies of *Ae. aegypti* identified a natural period of host-seeking inhibition where female mosquitoes are no longer attracted to host stimuli for three days after a blood meal [4](Figure 1). It has been speculated that this period of inhibition evolved as a defensive mechanism to protect females during egg development because *Ae. aegypti* feed during the day while potential hosts are alert [3]. During approximately three days of inhibition, eggs are developed and behavioral attraction switches from human hosts to stimuli associated with suitable egg-laying sites [5]. Only after females deposit eggs is attraction to host stimuli recovered, starting a new cycle of host-seeking, blood-feeding, inhibition, and egg-laying, known as the gonotrophic cycle [6]. This natural mechanism for regulating host-seeking behavior, if understood mechanistically, could inform novel strategies for combating the spread of mosquito-borne diseases.

At least two mechanisms for host-seeking inhibition have been proposed. First, abdominal distension triggered by blood ingestion is communicated through the ventral nerve cord to inhibit host attraction for approximately 24 hours [7](Figure 1B). Second, a humoral substance discovered in hemolymph at 48 hours after a blood meal is capable of suppressing host-seeking behavior when injected into non-blood-fed females [4](Figure 1B). In 1989, Matsumoto and colleagues identified Head Peptide-I (pERPhPSLKTRFa; pE: pyroglutamic acid; hP: hydroxyproline; and a: amidation) from mosquito head extracts as a candidate humoral factor mediating this suppression [8]. More recently, Head Peptide-I was also reported to be synthesized in the male-accessory gland and transferred to the female during copulation [9], although the behavioral relevance of this exchange is unknown. Further studies supported a role for Head Peptide-I in host-seeking regulation because titers of the peptide increased in the hemolymph during inhibition [10], and injection of synthetic Head Peptide-I into non-blood-fed females was sufficient to inhibit host-seeking behavior [10]. However, the mechanism by which Head Peptide-I alters mosquito behavior and the receptor through which it signals are unknown.

The aim of this study was to identify receptors that respond to Head Peptide-I as a means to clarify the mechanism for inhibition of host-seeking behavior following a blood meal. We carried out a cell-based calcium imaging assay to link candidate *Ae. aegypti* Neuropeptide Y-Like receptors to a panel of peptide ligands. We identified NPYLR1 as a candidate receptor for sNPF neuropeptides that showed lower sensitivity to Head Peptide-I. To study the *in vivo* role of NPYLR1 we used zinc-finger nucleases to generate *npylr1* mutant mosquitoes. Although NPYLR1 was the only receptor identified in our screen that responded to Head Peptide-I, *npylr1* mutants displayed no changes in feeding or inhibition of host-seeking behavior. Our interpretation is that other signaling mechanisms must exist for this important behavioral regulation that function even after removal of NPYLR1 activity.

## Methods

### Ethics statement

Work on vertebrate animals in this study was governed by and in compliance with the Animal Welfare Act (enforced by the USDA) and the Public Health Services Policy (enforced by the Office of Laboratory Animal Welfare) as well as New York State regulations. In addition, The Rockefeller University is an AAALAC accredited research facility committed to upholding the care standards provided by the 8th edition of the Guide for the Care and Use of Laboratory Animals. All blood-feeding procedures with mice were approved and monitored by The Rockefeller University Institutional Animal Care and Use Committee (approved protocol #11487). Work with normal human volunteers was conducted in compliance with the Declaration of Helsinki Principles. All blood-feeding and behavioral experiments with human volunteers were approved by the Institutional Review Board of The Rockefeller University Hospital (approved protocol LVO-0652). All human subjects gave their written informed consent to participate in these experiments.

### Mosquito maintenance

*Ae. aegypti* mosquitoes (Orlando strain) were maintained at 25–28°C with 70–80% relative humidity under a 14 hour light: 10 hour dark cycle (lights on 8 am). Eggs were hatched in de-oxygenated, deionized water containing powdered Tetramin tropical fish food (Tetra, Melle, Germany). Larvae were cultured in deionized water and fed Tetramin tablets as needed. Adults were given unlimited access to 10% sucrose solution. Adult females were blood-fed on mice for stock maintenance and on human arms and legs for isolation of mutants, egg-laying, and host-seeking behavior experiments.

### Bioinformatics

The 49 predicted class A rhodopsin-like GPCRs in the “peptide” and “orphan” receptor categories described in the *Ae. aegypti* genome publication [11] were analyzed for similarity to *D. melanogaster* and *An. gambiae* NPYLRs. From this group, eight candidate *npylr* genes were identified. Further analysis revealed that two of the eight were duplicate gene predictions that we combined to construct complete annotations for six candidate *npylr* genes. NCBI BLAST, Genewise (http://www.ebi.ac.uk/Tools/psa/genewise), and HMMER (http://hmmer.janelia.org/) bioinformatics tools were used to identify two additional *npylr* genes from published raw genomic sequence reads, returning the total candidate list to eight. All protein alignments and phylogenetic analyses were performed using default ClustalW and neighbor-joining tree-building methods in MacVector (http://www.macvector.com). Snake plots of predicted receptor topology were created using toppred (http://mobyle.pasteur.fr/).

A candidate Head Peptide gene from the tick, *Ix. scapularis*, was identified with tblastn searches of *Ae. aegypti* Head Peptide-I protein sequence against the trace reads archive available at NCBI in 2013 (ftp://ftp-private.ncbi.nlm.nih.gov/pub/TraceDB/ixodes_scapularis); trace identifier (TI Number): 1355069083, trace name: 1101271482505, center: J. Craig Venter Institute (http://gsc.jcvi.org/projects/msc/ixodes_scapularis/ixodes_scapularis/index.shtml).

### Molecular biology

RNA was isolated using RNeasy Mini Kits (Qiagen, Valencia, CA, USA) from various tissues as noted. DNA isolation from whole mosquitoes was carried out with the Qiagen DNAeasy Blood & Tissue Kit, with samples homogenized with 2 mm glass beads (Sigma Aldrich Cat#Z273627-1EA, St. Louis, MO, USA) using a Qiagen TissueLyzer II at 1800 rpm for 1 min. Unless otherwise noted, synthesis of cDNA was performed using SuperScript III Reverse Transcriptase (Invitrogen, Grand Island, NY, USA) and polymerase chain reactions (PCR) were performed using EMD Millipore KOD polymerase (Billerica, MA, USA). All DNA sequencing was performed by Genewiz (South Plainfield, NJ, USA).

### Receptor cloning

Full-length cDNAs for *Ae. aegypti npylr2*, *npylr5*, *npylr6*, and *npylr8* were obtained by reverse transcriptase coupled to the polymerase chain reaction (RT-PCR) with primers located at the 5′ and 3′ ends of the predicted open reading frame from purified *Ae. aegypti* female head and body RNA. Full-length cDNAs for *Ae. aegypti npylr1* (alleles *a*, *b*, *c*, *d*), *npylr3*, *npylr4*, *npylr7* (alleles *a* and *b*) were obtained by RT-PCR using Advantage 2 PCR polymerase (Clontech/Takara Bio, Mountain View, CA, USA) based on 5′ and 3′ sequences confirmed by rapid amplification of cDNA ends (RACE, Clontech/Takara Bio).

For both *npylr1* and *npylr7*, we isolated multiple alleles from the Orlando strain. These alleles contained sequence polymorphisms that produced changes to the protein coding sequence of the corresponding receptor as follows: NPYLR1A: D225E, 413ΔT, N487T relative to the genome reference NPYLR1B; NPYLR1B: genome reference sequence with GenBank accession KC439529 and found on Vectorbase supercontigs AAEL013505 and AAEL007924; NPYLR1C: D225E, N487T relative to the genome reference NPYLR1B; NPYLR1D: D225E, N487T, L495F relative to the genome reference NPYLR1B; NPYLR7A: genome reference sequence with Genbank accession KC439537 and associated with Vectorbase supercontigs AAEL008296 and AAEL015418; NPYLR7B: R14Q, L161F, Y189H, R250Q relative to genome reference NPYLR7A. This allele also has an insertion of a T residue at amino acid position 22. The numbering of polymorphisms C-terminal to this residue refers to NYPLR7A numbering.

*An. gambiae snpfr* was amplified from RNA derived from antennal tissue of the G3 strain based on published sequences by Garczynski and colleagues [12]. *D. melanogaster snpfr* clone GH23382 was obtained from the *Drosophila* Genomics Resource Center (https://dgrc.cgb.indiana.edu/). *Ix. scapularis I.sca-npylr1a* and *I.sca-npylr1b* were identified in the draft *Ix. scapularis* genome (IscaW1.2) in Vectorbase (http://www.vectorbase.org) using blastp. Both receptor cDNAs were cloned using RT-PCR from whole tick tissue provided by Rick Ostfeld (Cary Institute, Millbrook, NY). We were unable to determine if *I.sca-npylr1a* and *I.sca-npylr1b* are encoded by two distinct genes or are variant alleles produced by the same gene.

The sequences of all oligonucleotide primers used in this study are listed below.

All receptor cDNAs were cloned into Invitrogen's TOPO-TA Cloning system for propagation in TOP10 or DH5alpha cells. For the cell-based assay, all receptor cDNAs were recloned into the XhoI-NotI sites of the mammalian expression vector pME18S [13] except for *A.gam-snpfr* and *D.mel-snpfr*, which were cloned into EcoRI-NotI sites of the same vector.

Genbank accession numbers for the cDNAs used in this study are as follows: *Ae. aegypti* genes: *npylr1a* KC439528, *npylr1b* KC439529, *npylr1c* KC439530, *npylr1d* KC439531, *npylr2* KC439532, *npylr3* KC439533, *npylr4* KC439534, *npylr5* KC439535, *npylr6* KC439536, *npylr7a* KC439537, *npylr7b* KC439538, and *npylr8* KC439539.

*Ix. scapularis* genes: *I.sca-npylr1a* KC439540 and *I.sca-npylr1b* KC439541.

### Primers

The following oligonucleotide primers used in this study were synthesized by Integrated DNA Technologies (Coralville, IA, USA): Primers for receptor cDNA cloning: *A.aeg-npylr1a,b,c,d* forward, 5′-ATGGCCATAACGATGTCATCACG-3′; *A.aeg-npylr1a,b,c,d* reverse, 5′-TTACAGTATCTCCGGCAGCTTGG-3′; *A.aeg-npylr2* forward, 5′-ATGCTGGCAAGTACCGCTAAGAC-3′; *A.aeg-npylr2* reverse, 5′-TTACAAACGTGTAATGTCTTCTTGGAAGC-3′; *A.aeg-npylr3* forward, 5′-ATGAAGTCCAAGGAGACCGCGTCGGATGC-3′; *A.aeg-npylr3* reverse, 5′-CTCGCCCGTAATCTTTGGCACCGC-3′; *A.aeg-npylr4* forward, 5′-CGTTGTCAGCTTCGACGATGAGTGT-3′; *A.aeg-npylr4* reverse, 5′-CGCCAGGAAACGTGCAGCTTCG-3′; *A.aeg-npylr5* forward, 5′-ATGAGCGGCGCGCCATTCACGGTC-3′; *A.aeg-npylr5* reverse, 5′-TCACCGTAGCAGGGACGTTTCCGT-3′; *A.aeg-npylr6* forward, 5′-CACGCCACAATGGATTACCC-3′; *A.aeg-npylr6* reverse, 5′-CATCACTTGAACAGGATCCGC-3′; *A.aeg-npylr7a,b* forward, 5′-GCGATGAACTTCACTGCCGAGTT-3′; *A.aeg-npylr7a,b* reverse, 5′-CTACAACCCCTTCCGGCACCACT-3′; *A.aeg-npylr8* forward, 5′-ATGGACGTGGTCCTGTCCAGGCTG-3′; *A.aeg-npylr8* reverse, 5′-TCACGGCATGAGCTCGGTAAGC-3′; *Agam-snpfr* forward, 5′-TTATAGAATAGCGGGCACTTTCGAGTC-3′; *Agam-snpfr* reverse, 5′-GACGCCTCGGAATGCTGACG-3′; *I.sca-npylr1a* forward, 5′-AACCCAAGCTTGTTCAATCC-3′; *I.sca-npylr1a* reverse, 5′-ATGCTGACATCTGGGGGTAG-3′; *I.sca-npylr1b* forward, 5′-TTCTTGCAGATGTCGGATCA-3′; *I.sca-npylr1b* reverse, 5′-TTTCTCCATGTTGCAGTGCT-3′; *D.mel-snpfr76f* forward, 5′-ATGGCCAACTTAAGCTGGCTGAG-3′; *D.mel-snpfr76f* reverse, 5′-CCTATCTCAGTTGATTCGCCTC-3′. Primers for qPCR: *A.aeg-npylr1* forward, 5′-GCTATCTGCTACATCTGTGTGTCAA-3′; *A.aeg-npylr1* reverse, 5′-GTCCGAGTAGAAGTCGTTGCTCAT-3′; *A.aeg-rpl8* forward, 5′-TCACTGCCCACACCAAGAAGCG-3′; *A.aeg-rpl8* reverse, 5′-CGGCAATGAACAACTGCTTGCG-3′. Primers for *A.aeg-npylr1* homologous recombination arms: Left arm forward, 5′-TGCTGGCGTTACGGCAAACTGATTC-3′; Left arm reverse, 5′-GAACGTCACATTAACAGCGTCGCTG-3′; Right arm forward, 5′-GGTCAAGCCTTGATGCAGGACAATAC-3′; Right arm reverse, 5′-AGTATCTCCGGCAGCTTGGTGTCG-3′. Primers for non-homologous end-joining genotyping: *A.aeg-npylr1* forward, 5′-CGGAACTTACGAAGCATTCAGCGAC-3′; *A.aeg-npylr1* reverse, 5′-GAACACTACGTAGCATACCAACACG-3′. Primers for homologous recombination genotyping: *A.aeg-npylr1* forward, 5′-TAATCGTGTGGACTAGAAGAGGG-3′; *A.aeg-npylr1* reverse, 5′-AGCTCTTCGCAGTAGAATGTACG-3′.

### HEK293T cell-based calcium imaging assay

HEK293T cells were cultured using standard protocols in a Thermo Scientific FORMA Series II – Water Jacketed CO_2_ incubator (Waltham, MA, USA). Lipofectamine 2000 (Invitrogen) was used for transient transfection of 1 µg pME18S>Candidate receptor and 1 µg pME18S>murine G_q_α15 [14]. Transfected cells were loaded with the calcium sensitive dye Fura-2 (Invitrogen) according to product instructions. Cells were imaged on a Nikon Eclipse TE-2000-U microscope (Melville, NY, USA) using fluorescent excitation from a Lambda DG-4 (Sutter Instruments Co., Novato, CA, USA). Bath application of ∼300 µl solutions containing peptides diluted in Ringer's solution (0.14 M NaCl, 5.6 mM KCl, 5 mM HEPES, 2 mM pyruvic acid, 2 mM CaCl_2_, 2 mM MgCl_2_, 0.15% Glucose, 1.25 mM KH_2_PO_4_, pH 7.4) were delivered using a diaphragm pump (Gilson Minipuls3, Middleton, WI, USA) with real-time recording and analysis in Metafluor software (Version 7.1.2.0; Molecular Devices, Sunnyvale, CA, USA). Data from a complete peptide dilution series were collected from eight responding cells within each plate of three independent transfections. Data were normalized by setting the highest fluorescent response within each experiment to 100%.

The variant alleles of NPYLR1 (alleles A–D) and NPYLR7 (alleles A–B) were tested in the cell-based assay as follows: NPYLR1A, NPYLR7A, and NPYLR7B were tested against the entire panel of peptides. NPYLR1B, NPYLR1C, and NPYLR1D were tested against Head Peptide-I and sNPF-3 only.

### Behavior assays

#### Uniport olfactometer

The uniport olfactometer was custom built by Vadim Sherman in the Rockefeller University High Energy Physics Instrument Shop. Approximately 15–25 female mosquitoes aged 5 to 14 days were placed within loaders, which are small plastic cylinders with mesh covering both openings and a sliding door on one end obtained from the World Health Organization Vector Control Research Unit (Penang, Malaysia). Sliders were attached to one end of a 1 m long plastic tube (19 cm diameter) that led to an “attraction trap” (14 cm long, 5 cm diameter), followed by a sealed chamber into which a human volunteer inserted a hand or forearm (see image in Figure 1A for the configuration with a hand stimulus). Humidified room air was carbon-filtered (Donaldson Ultrac-A, Bloomington, MN), supplemented to a final concentration of 5% CO_2_ using flow-meters (Cole Parmer, catalogue #023-92-ST, Vernon Hills, IL), and passed over the hand or forearm of the volunteer into the olfactometer at 3.8 L/min. Mosquito loaders were attached to the olfactometer and given 5 minutes to acclimatize to air-flow prior to a 5 minute host-seeking test. Mosquitoes were scored as attracted if they flew upwind through the 1 m tube and into the “attraction trap” within the allotted time.

#### Locomotor activity

Activity was monitored using LAM25 locomotor activity monitors (Trikinetics Inc., Waltham, MA, USA). 5 to 14 day-old sugar-fed female mosquitoes were individually placed into glass tubes (25 mm diameter, 125 mm long). A 10% sucrose-soaked cotton plug sealed one end of the glass tube to serve as a food source for the mosquito during the experiment. The vials were inserted into the monitor and housed within a Digitherm incubator (Tritech Research Inc., Los Angeles, CA, USA) set to 25°C and 70–80% relative humidity under a 14 hour light: 10 hour dark cycle (lights on at 8am). Infrared beam breaks triggered by the movement of the mosquito were recorded continuously and tabulated into 1 min bins. Bins with 60 or more beam breaks per minute (>1/sec) and trials with 2000 or more beam breaks per day were excluded from the analysis.

#### Egg-laying

5 to 14 day-old female mosquitoes were blood-fed on a human arm or leg for 15 minutes. 72–96 hours after feeding, individual mosquitoes were placed in plastic fly vials (25 mm diameter, 95 mm long) containing 5 ml water and a Whatman filter paper (55 mm diameter, GE Healthcare, Buckinghamshire, UK) folded into a cone. The filter paper became moist from the water and acted as a substrate for females to lay eggs. At 144 hours post blood-meal, Whatman papers were removed and eggs were manually counted using a Nikon SMZ1500 dissecting microscope.

#### CAFE assay

This assay was adapted for mosquitoes from the CAFE assay of Ja and colleagues used to analyze feeding behavior in *Drosophila melanogaster* [15]. Female mosquitoes aged 5 to 14 days were fasted from sugar for the indicated amount of time. After fasting, five mosquitoes were transferred into a plastic fly vial (25 mm diameter, 95 mm long) containing a cotton plug pierced by a 5 µl calibrated pipet (VWR International, catalogue #53432-706) filled with a known volume of 10% sucrose. After 2 hours, the pipets were removed and the change in sucrose volume was measured by noting the change in level of the liquid meniscus using a ruler. A control vial was set up to measure the loss of volume due to evaporation for each experiment, which was corrected for all test vials.

#### BUFFET assay

Approximately 50 female mosquitoes were fasted for 48 hours in a mosquito bucket cage. 96-well plates (Biorad, catalogue #HSP-9661) filled with 200 µl 10% sucrose+0.02% fluorescein (Sigma Aldrich, catalogue #16377) were placed inside the cage and mosquitoes were allowed to feed for 2 hours. Afterwards, mosquitoes were frozen at −20°C and 16 individuals were randomly selected and prepared for fluorescent measurement within 4 hours (see fluorescent measurement methods below).

#### Membrane feeding assay

Glass feeders (20 mm Glass Jacketed Membrane Feeder, Chemglass Life Sciences, Vineland, NJ, USA) were filled with 400 µl defibrinated sheep blood (Hemostat Laboratories, Dixon, CA, USA)+0.02% fluorescein. The larger opening of the feeder was covered with two layers of Parafilm stretched thin to facilitate puncturing by the mosquitoes. The other end was also sealed with Parafilm to prevent spillage. Fifteen mosquitoes aged 5 to 14 days were placed into 6 separate small cups (16 oz SOLO paper cups, www.webstaurantstore.com) sealed on top by white mesh and loaded into a sealable plastic container. Carbon-filtered air was pumped into the plastic chamber through a flypad (8.9×12.7×1 cm; catalogue #MINJ-DROS-FP; tritechresearch.com, Los Angeles, CA). One blood-filled feeder was placed on top of each cup and connected by hosing to a 37°C water bath and left for 5 minutes so that mosquitoes could acclimatize. To begin the experiment, water pumps began circulation of 37°C water through the membrane feeder hosing and the air stream was supplemented to a final concentration of 5% CO_2_ to activate the mosquitoes. After 15 minutes, the small cups were frozen at −20°C and prepared for fluorescent measurement within 4 hours (see fluorescent measurement methods below).

#### Host-seeking behavior

Large groups of ∼300 female mosquitoes per genotype were fed simultaneously on the arms and legs of human volunteers (typically at 1pm). Animals that had not fed to completion were visually identified under cold anesthesia and discarded. Approximately 15 to 25 blood-fed mosquitoes were transferred to loaders 4 hours before testing at 0 (before blood), 24, 48, 72, 96, and 120 hours post-blood meal in the uniport olfactometer. Access to egg laying substrate was provided after 72 hours and egg-laying was typically finished by ∼110 hours. Mosquitoes were behaviorally tested for only one time point and then discarded.

### Fluorescent measurements

Frozen mosquitoes were loaded individually into wells of a 96-well plate (Biorad, catalogue #HSP-9661) containing 100 µl phosphate-buffered saline (PBS) (prepared as a 1× solution from 10× PBS without Ca^2+^ or Mg^2+^; Lonza, BioWhittaker; Walkersville MD) plus one 2 mm glass bead (Sigma Aldrich, Cat#Z273627-1EA) and covered with PCR Sealers (BioRad, catalogue #MSB1001, Hercules, CA, USA). Control wells containing 1, 2, 3, 4, 5, 6, 7, 8, 9, 10, 15, 20, 25 µl of 0.02% fluorescein were combined with unfed control female mosquitoes to create a reference dilution curve. Plates were homogenized using a Qiagen TissueLyzer II at 1800 rpm for 1 minute. Homogenized plates were taken to the Rockefeller University High-Throughput Screening Resource Center, where 15 µl of homogenized solution from each well was transferred to a 384 well plate (Greiner Bio One, catalogue #784201, Monroe, NC, USA) alongside 15 µl of a 1∶10 dilution in PBS dispensed using a Thermo Multi-Drop Combi and Perkin Elmer JANUS Mini (Waltham, MA, USA). Samples were vortexed briefly and fluorescent intensity for each well was measured using a Biotek Synergy NEO plate reader (Winooski, VT, USA). Using the reference dilution curve, fluorescent measurements were converted back to volume (µl) of solution ingested.

### Peptide synthesis

Head Peptide-I (pERPhPSLKTRFa), Head Peptide-III (pERPPSLKTRFa), and Head Peptide-I [Cys10] (pERPhPSLKTRC) were synthesized by the Rockefeller University Proteomics Resource Center. sNPF-1 (KAVRSPSLRLRFa), sNPF-1(4-11) (SPSLRLRFa), sNPF-2+4 (APQLRLRFa), and sNPF-3 (APSQRLRWa), NPF (SFTCARPQDDPTSVAEAIRLLQELETKHAQHARPRFa), human NPY (YPSKPDNPGEDAPAEDMARYYSALRHYINLITRQRYa), and human PYY (IKPEAPGEDASPEELNRYYASLRHYLNLVTRQRYa) were synthesized by Bachem Bioscience Inc. (King of Prussia, PA, USA).

### Peptide injections

Solutions of Head Peptide-I, sNPF-3 and Head Peptide-I [Cys10] were dissolved in saline (0.1M NaCl, 4 mM KCl, 2 mM CaCl_2_) at a concentration of 4 and 10 mM for injection. ∼20 female mosquitoes aged 5 to 14 days were anesthetized on ice for 3 min, moved individually onto a chill-plate (BioQuip catalogue #1429, Rancho Dominguez, CA, USA), and injected with 200 nl of desired solution using a Drummond Nanoject II (Drummond catalogue # 3-000-204, Broomall, PA, USA) attached to 3.5″ needles (Drummond, catalogue #3-000-203-G/X) shaped on a needle puller (Sutter Instruments Co., Model P-97). Solutions were injected into the abdomen by piercing the second abdominal tergite from the ovipositor. Injected mosquitoes were placed in loaders and allowed one hour to recover from injection before being tested for host-seeking behavior in the uniport olfactometer.

### Quantitative PCR (qPCR)

RNA was purified from whole bodies of female mosquitoes before and 24, 48, and 72 hours after blood-feeding. Purified RNA was converted to cDNA and aliquoted at 250 ng/µl RNA equivalents for each qPCR reaction. Reactions were prepared as instructed by BioRad iQ SYBR Green SuperMix (catalogue #170-8882) in BioRad iQ 96-well Plates (catalogue #223-9441) covered with BioRad Microseal “B” Adhesive Seals (catalogue #MSB-1001) to be run on a iQ5 iCycler (BioRad). Primers were designed to meet the following criteria: 90–110% efficiency and an R^2^ above 0.98 in control reactions. Three biological replicates of each sample were performed in triplicate for every condition.

### Targeted mutagenesis with zinc-finger nucleases

Zinc-finger nucleases targeting *Ae.aegypti npylr1* were designed using the CompoZr Custom ZFN technology by Sigma-Aldrich Life Science (St. Louis, MO, USA). Genetic Services Inc. (Cambridge, MA, USA) injected purified *npylr1* ZFN mRNA into ∼3000 pre-blastoderm stage *Ae. aegypti* Orlando embryos (three batches of 1000, 800, and 1200 eggs each) at a concentration of 200 ng/µl plus a homologous recombination vector at 850 ng/µl using embryo preparation methods described previously [16]. The homologous recombination vector contained 1319 and 1451 bp of homology to the left and right flanking sequence of the ZFN cut site and were obtained by PCR reactions from *Ae. aegypti* Orlando genomic DNA. Left and right arms were cloned into SalI and NotI sites, respectively, of the homologous recombination donor plasmid pSL1180-HR-PUbECFP (a gift of Conor McMeniman and deposited in Addgene, plasmid number 47917) that contains the *Ae. aegypti polyubiquitin* promoter [17] driving expression of ECFP, for a total insert size of 2565 bp.

36% of injected embryos hatched and were sexed as male and female prior to eclosion. 94% of the hatched individuals developed into adults and each injection batch was inter-crossed after reaching sexual maturity (∼2 days). Each of the three inter-crossed groups was considered to contain independent ZFN events. Potential mutants carrying the *polyubiquitin*-ECFP cassette were isolated by aliquoting 3-day old generation 1 (G1) larva into 96-well plates (VWR International, catalogue #29444-018) and screening for ECFP fluorescence on a Nikon SMZ1500 excited by an Intensilight C-HGFI. Of an estimated 55,000 total larvae screened, 27, 6, and 18 larvae were ECFP positive in each respective batch.

Non-homologous end-joining mutants were isolated a few months later from a small number of unhatched eggs stored from the third injection batch of G1 mosquitoes. Seven G1 larvae hatched and were outcrossed to wild-type *Ae. aegypti* Orlando. After successful mating, genomic DNA was prepared from each of the seven individuals and *npylr1* PCR amplicons were sequenced for ZFN activity. Small deletions were detected in two individuals. PCR clones for the two positive individuals were Sanger sequenced by Genewiz to confirm 4 and 8 bp deletions, respectively.

For each non-homologous end-joining mutant, G1 individuals were inter-crossed for four generations to establish homozygous lines. Genotyping of individual non-homologous end-joining mutants was carried out by Genewiz using a capillary gel electrophoresis DNA analyzer (ABI3730xl, Applied Biosystems, Carlsbad, CA, USA) to analyze PCR amplicons from 6-FAM fluorescently labeled primers spanning the *npylr1* ZFN cut site. Data were analyzed using Peak Scanner software (Applied Biosystems). Homologous recombination mutants were out-crossed to *Ae. aegypti* Orlando for five generations prior to establishing homozygous lines. Mutant individuals carrying the integrated homologous recombination cassette were genotyped by gel electrophoresis of PCR amplicons spanning the inserted DNA. Heterozygous control lines for behavioral experiments were generated by crossing homozygous mutant lines to the *Ae. aegypti* Orlando strain, which expresses a mixture of the four functionally equivalent *npylr1* alleles.

### Statistics

Statistical tests were performed as indicated in each figure legend using Graph Pad PRISM (La Jolla, CA, USA) except for statistical analysis of qPCR results, which also used BioRad iQ5 software.

## Results

### NPYLR1 identified as a receptor sensitive to Head Peptide-I and sNPFs

**Figure 2:**
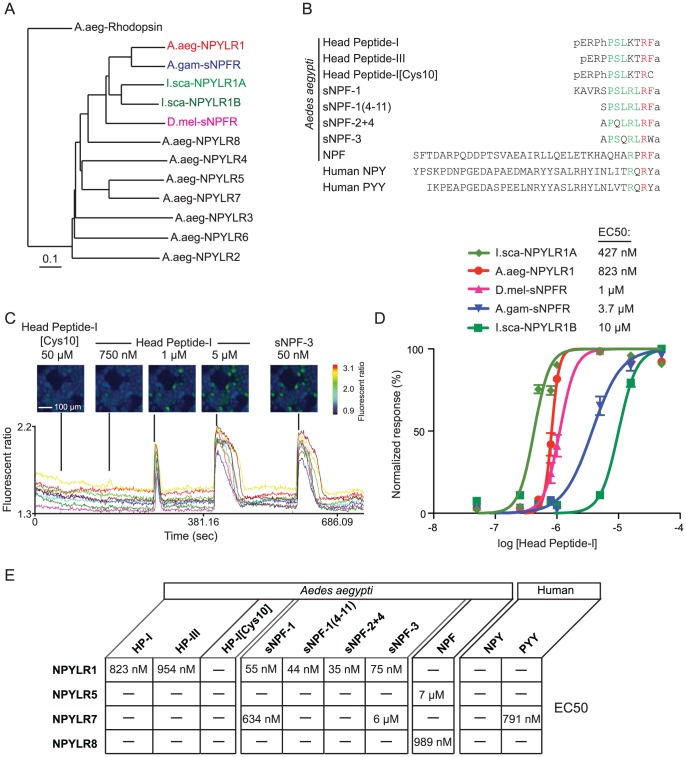
Deorphanization of NPY-Like receptors. (A) Phylogram depicting the genetic relationships of eight *Ae. aegypti* NPY-Like Receptors (NPYLR1, red; NPYLR2-8, black) as well as NPYLR1 homologues *An. gambiae*-sNPFR (blue), *Ix. scapularis* I.sca-NPYLR1A and I.sca-NPYLR1B (light and dark green), and *D. melanogaster*-sNPFR (magenta). (B) Peptides used in the HEK293T cell-based calcium imaging assay displayed with single letter amino acid codes and these variants: pE: pyroglutamic acid, hP: hydroxyproline, a: amidation, sNPF: *Ae. aegypti* short-Neuropeptide-F, NPF: *Ae. aegypti* Neuropeptide-F, Human NPY: Neuropeptide Y, Human PYY: Peptide YY. (C) Top: Representative images of HEK293T cells transiently transfected with *Ae. aegypti npylr1a*, illustrating calcium flux after application of indicated peptides. Bottom: Individual traces of fluorescent ratios representing Ca^2+^ flux for the same HEK293T cells following application of the indicated peptides. (D) Head Peptide-I dose-response curves and EC50 values for NPYLR1 homologues in *Ix. scapularis*, *An. gambiae*, and *D. melanogaster*. (E) Summary of results for the cell-based calcium imaging screen. NPYLR2, NPYLR 3, NPYLR 4, and NPYLR 6 did not respond to any peptides tested (HP = Head Peptide).

Head Peptide-I shows sequence similarity to short neuropeptide-F peptides (sNPFs) that have been implicated in feeding behaviors [18]–[20] and are known to signal through Neuropeptide Y (NPY)-Like Receptors (NPYLRs) [21]. We identified eight *npylr* genes in the *Ae. aegypti* genome (Figure 2A), all of which are orphan receptors that have not been linked to a candidate peptide ligand. To deorphanize these receptors and identify those that respond to Head Peptide-I, we transfected each into HEK293T cells along with murine G_q_α15, a promiscuous Gα subunit capable of coupling diverse G protein-coupled receptors to a phospholipase C stimulated calcium signaling pathway [14]. We screened each receptor in a cell-based calcium imaging assay for sensitivity to a panel of *Ae. aegypti* peptides including Head Peptide-I [8], [10], behaviorally inactive Head Peptide-III [10], inactive Head Peptide-I [Cys10] [10], sNPF-1 [22], sNPF-1(4-11) [22], sNPF-2+4 [22], sNPF-3 [22], and NPF [22](Figure 2B). We also used human Neuropeptide Y (NPY) and Peptide YY (PYY) [23](Figure 2B). Cells transiently co-transfected with a given receptor and G_q_α15 were loaded with the calcium-sensitive dye Fura-2 and imaged in response to bath application of peptides (Figure 2C, D, and E).

Of the eight receptors screened, NYPLR1 was most sensitive to all four sNPFs (EC50: 35–75 nM), but also responded to Head Peptide-I, albeit at much higher concentrations (EC50: 823 nM). Head Peptide-I [Cys10] evoked no response at concentrations up to 50 µM (Figure 2C). The *Ae. aegypti* Orlando strain expresses four *npylr1* alleles, which were functionally indistinguishable in the cell-based assay (data not shown).

*Ae. aegypti* NPYLR1 is homologous to the sNPF receptors (sNPFR) of *D. melanogaster* [21], [24] and *An. gambiae* [12]. Contrary to previous reports in *An. gambiae* [12], we found that NPYLR1 orthologues from both insects responded to *Ae. aegypti* Head Peptide-I (Figure 2D), despite no evidence for a Head Peptide gene in either insect. Both orthologues were also reported to lack sensitivity to Head Peptide-III [12], [21], [24], but we found that NPYLR1 was equally sensitive to both Head Peptide-I and Head Peptide-III. We have no explanation for these discrepancies, but note that since *D. melanogaster* and *An. gambiae* lack a Head Peptide gene, the physiological relevance for the response or non-response of these orthologues to Head Peptides is unclear.

We identified a candidate Head Peptide gene in the incomplete genome of the Lyme disease vector, *Ixodes scapularis*, which like *Ae. aegypti* displays intermittent blood-feeding [25], [26]. The candidate *Ix. scapularis* Head Peptide gene would encode two copies of a pro-peptide with sequence ERPPPQKIRF, which is 70% similar and 60% identical to the pro-peptide form of *Ae. aegypti* Head Peptide-I: QRPPSLKTRF. We did not validate this prediction by RNA analysis in ticks nor did we functionally test whether this peptide, predicted solely based on *in silico* analysis, exists *in vivo* or would activate any of the NPYLRs tested here. We found no evidence for Head Peptide gene candidates in any other currently available genome except *Aedes aegypti*.

Because ticks have an apparent Head Peptide gene, we searched the genome for NPYLR1 orthologues and found two candidate genes. We cloned both tick NPYLR1 genes and demonstrated that each was sensitive to *Ae. aegypti* Head Peptide-I (Figure 2D). These results are the first evidence for Head Peptide-I signaling outside of *Ae. aegypti*.

In summary, four of eight *Ae. aegypti* NPYLRs responded to one or more of the peptide ligands (Figure 2E). We examined two *npylr7* alleles, and while NPYLR7A did not show any responses, NPYLR7B responded to three of ten peptides tested. The four NPYLRs that failed to respond to our panel of peptides (NPYLR2, NPYLR3, NPYLR4, and NPYLR6) may respond to other ligands not tested in our assay or may not be functionally expressed in our assay.

**Figure 3:**
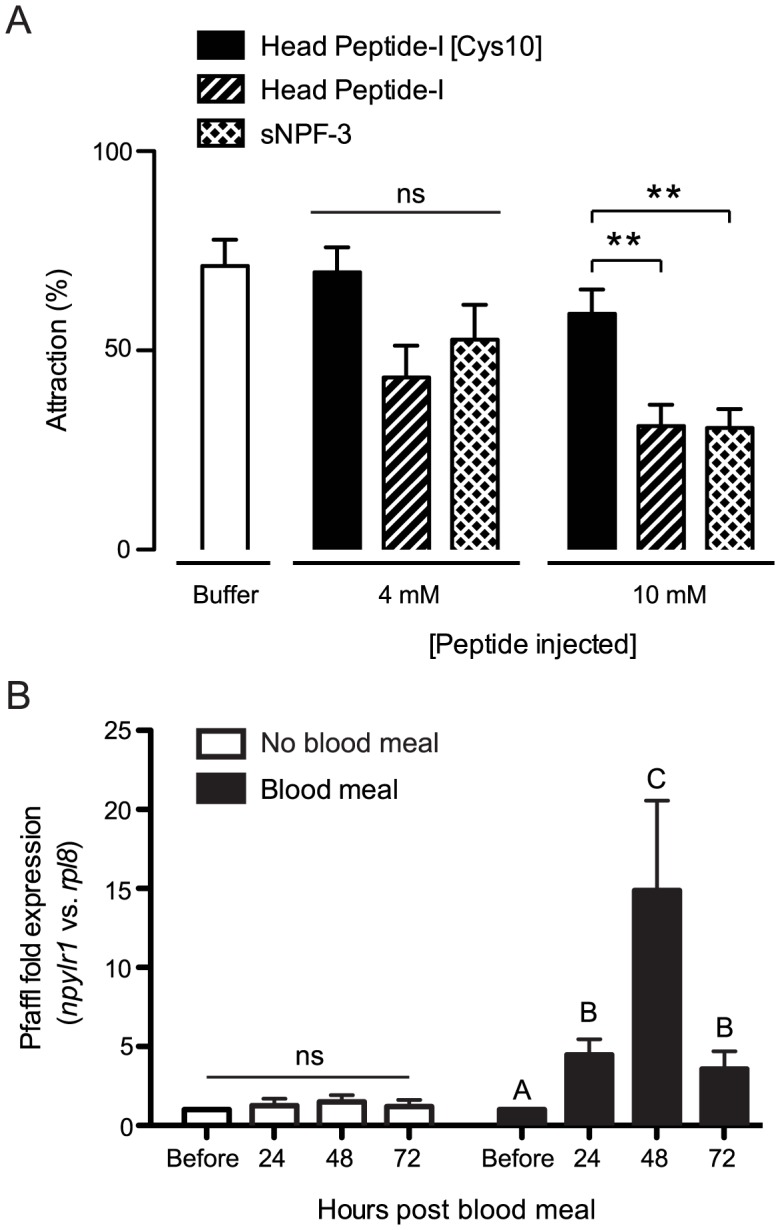
Injection of Head Peptide-I or sNPF-3 inhibits host-seeking behavior and *npylr1* expression is up-regulated after blood-feeding. (A) Percent attraction of non-blood-fed female mosquitoes to human host stimuli after injection of buffer (open bar) or 4 and 10 mM of the indicated peptide. (n = 6; ∼20 females per trial, ANOVA with Bonferroni correction for multiple comparisons; ** = p<0.01). (B) qPCR analysis of *npylr1* transcripts in total RNA extracted from female whole body tissue at the indicated times after a blood meal. (n = 3, ANOVA-Tukey's Multiple Comparison of ΔCt values). All data are plotted as mean±SEM.

With functional evidence that NPYLR1 responds to both Head Peptide-I and sNPFs, we tested whether sensitivity of the receptor to sNPFs indicated a role for these neuropeptides in host-seeking inhibition. To begin, we replicated previously published experiments that reported Head Peptide-I inhibition of host-seeking behavior by injection into non-bloodfed females [9] (Figure 3A). We then found that injection of sNPF-3 also inhibited host-seeking behavior in non-blood-fed females, whereas control injections of buffer or inactive Head Peptide-I [Cys10] had no effect (Figure 3A).

Given earlier observations that Head Peptide-I titers increase in hemolymph at 48 hours after blood-feeding [10], we asked if *npylr1* gene expression was similarly controlled by feeding state. Using qPCR analysis of RNA derived from female whole body tissue, we found that expression of *npylr1* is up-regulated for at least three days following a blood meal and peaks with a 15-fold increase at 48 hours (Figure 3B). This peak in *npylr1* expression is coincident with the time of maximal host-seeking inhibition (Figure 1B).

### Targeted mutagenesis of *npylr1* with zinc-finger nucleases

To ask if NPYLR1 is necessary for blood-feeding-induced host-seeking inhibition, we generated *npylr1* null mutant mosquitoes using targeted mutagenesis with zinc-finger nucleases (ZFNs). ZFNs are fusion proteins of a sequence-specific DNA binding protein and a *fokI*-nuclease that induces mutagenic double-stranded breaks [27], [28]. To prepare for mutagenesis, we clarified the gene structure of *npylr1*. The current draft of the *Ae. aegypti* genome (AaegL1.4) has two gene entries for *npylr1*, suggestive of a gene duplication (AAEL013505 and AAEL007924). We used Southern blotting and PCR-based genotyping to confirm that these two annotated genes are in fact two alleles of a single *npylr1* gene locus that show normal Mendelian segregation (data not shown).

**Figure 4:**
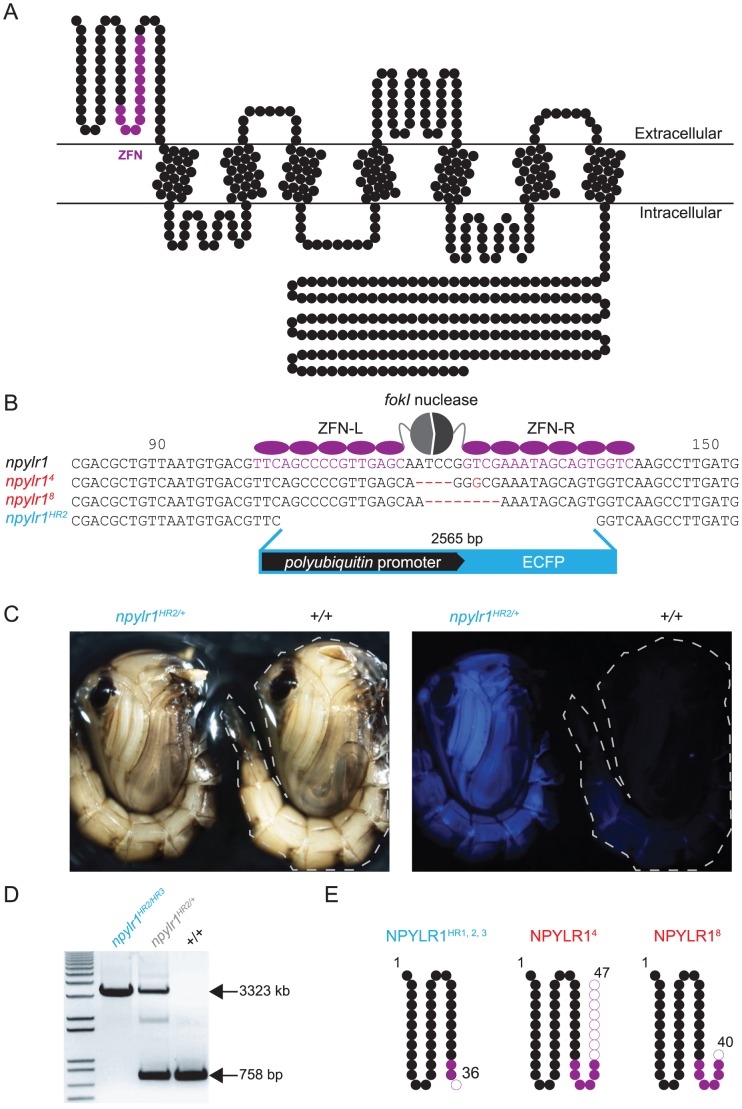
Targeted mutagenesis of *npylr1* using zinc-finger nucleases. (A) Snake plot of predicted NPYLR1 membrane topology with the corresponding protein region encoded by the *npylr1* zinc-finger nuclease targeted DNA sequence colored in purple. (B) Illustration of the zinc-finger nuclease target region in the *npylr1* gene with corresponding mutations generated by non-homologous end-joining (*npylr1^4^* and *npylr1^8^*, red) and integration of a fluorescent marker by homologous recombination (*npylr1^HR2^*, cyan). (C) Photographs of *npylr1^HR2/+^* and wild-type mosquito pupae illustrating broad expression of the ECFP marker. (D) PCR verification of homologous recombination into the *npylr1* locus of the 2565 bp *polyubiquitin*>ECFP cassette. (E) Snake plots of predicted protein truncations for *npylr1* mutant alleles. Filled purple circles indicate ZFN target region and outlined purple circles indicate resulting frame-shift mutations.

ZFNs targeting the 5′ end of the coding sequence encoding the N-terminal region of NPYLR1 (Figure 4A) were injected into wild-type mosquito embryos together with a DNA vector that would act as a template for repair by homologous recombination (HR). Three independent mutation events (*npylr1^HR1^*, *npylr1^HR2^*, *npylr1^HR3^*) were confirmed by integration of a marker cassette containing a broadly expressed fluorescent protein into the *npylr1* locus (Figure 4B and 4C). Southern blotting and PCR analysis confirmed each event to be a single integration at the *npylr1* locus (Figure 4D and data not shown). Additional injected progeny were analyzed to isolate two new alleles lacking the ECFP cassette and produced by error-prone non-homologous end-joining, *npylr1^4^* and *npyl1^8^*, with deletions of 4 bp and 8 bp respectively (Figure 4B). All five mutant alleles are predicted to create truncated NPYLR1 protein (Figure 4E). Using these homozygous mutants, we generated heteroallelic *npylr1* mutant combinations to control for independent background mutations for behavioral analysis.

### Locomotor and egg-laying behavior of *npylr1* mutants

**Figure 5:**
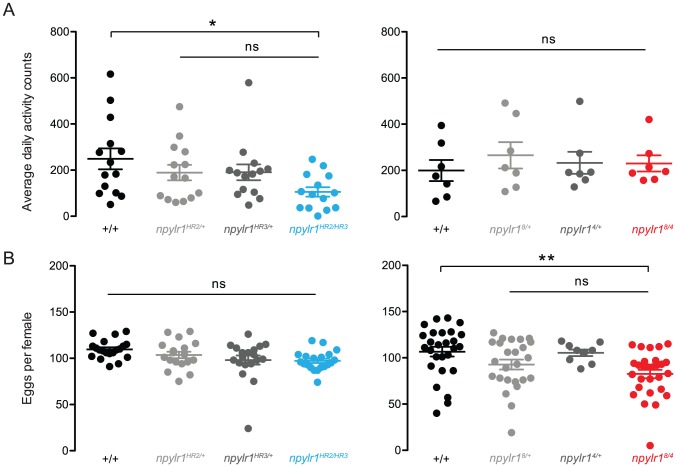
Normal locomotor behavior and egg-laying in *npylr1* mutants. (A) Average daily activity counts for individual female *npylr1^HR2/HR3^* mutants (left, n = 14) and *npylr1^8/4^* mutants (right, n = 7). (B) Eggs deposited per individual female *npylr1^HR2/HR3^* (left, n = 18–20) and *npylr1^8/4^* (right, n = 9–28). All data are plotted as mean±SEM. 1-Way ANOVA with Bonferroni correction for multiple comparisons. * = p<0.05; ** = p<0.01, ns = not significant.

As a likely receptor for neuromodulation, NPYLR1 may be involved in broader physiological roles such as motor coordination or egg development [29]. We found that locomotor activity of *npylr1^8/4^* was indistinguishable from control lines (Figure 5A right). *npylr1^HR2/HR3^* showed a decrease in average daily activity in comparison to wild-type but was not significantly different from either *npylr1^HR2/+^* or *npylr1^HR3/+^* controls (Figure 5A left). Based on these data, we conclude that locomotor activity is generally normal in *npylr1* mutants.

Quantification of deposited eggs from individual females blood-fed to completion on a human arm showed no differences in *npylr1^HR2/HR3^* (Figure 5B left). A slight decrease was observed for *npylr1^8/4^* compared to wild-type, but not to *npylr1^8/+^* or *npylr1^4/+^* controls (Figure 5B right). Based on these results, we conclude that egg-laying is unaffected in *npylr1* mutants.

### Sugar and blood feeding of *npylr1* mutants

Beyond acting as a putative modulator of blood-feeding-induced host-seeking behavior, sNPFs and/or Head Peptide-I may communicate a general internal fed state [18]. Female mosquitoes do not feed only on blood, but also on floral nectars to satisfy metabolic needs [30]. It is not known whether mosquitoes independently regulate sugar- and blood-feeding, so we examined both in *npylr1* mutants.

**Figure 6:**
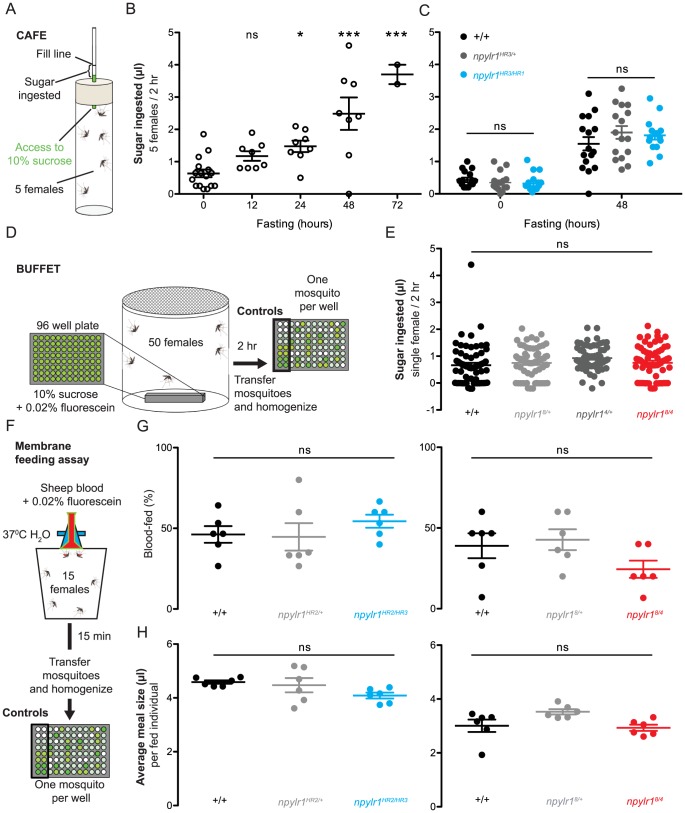
Fasting response, sugar-feeding and blood-feeding are normal in *npylr1* mutants. (A) Diagram of the CAFE assay. (B) Volume of sugar ingested for female mosquitoes in the CAFE after fasting for 0, 12, 24, 48, and 72 hours (n = 8–16, except 72 hours, n = 2; 5 mosquitoes per trial. Mean±SEM. 1-way ANOVA compared to 0 hours with Dunnett's multiple comparison test. * = p<0.05, *** = p<0.001, ns = not significant). (C) Sugar ingested by the indicated genotypes at 0 and 48 hours after fasting (n = 16; 5 females per trial. Mean±SEM, 2-way ANOVA with Bonferroni correction for multiple comparisons). (D) Diagram of the BUFFET assay. (E) Sugar ingested by the indicated genotypes at 48 hours after fasting (n = 64; 16 females tested in each of 4 independent trials). (F) Diagram of the membrane feeding assay. (G) Percent of mosquitoes for the indicated genotypes that blood-fed in the membrane feeding assay (n = 6, 15 females per trial). (H) The average blood meal sizes for the indicated genotypes. (n = 6, 1–12 females confirmed as fed per trial). Data in E, G, H are plotted as mean±SEM, 1-way ANOVA with Bonferroni correction for multiple comparisons.

We studied sugar-feeding using the CAFE (Capillary Feeder) assay adapted from a similar assay originally designed for *D. melanogaster* [15]. In this assay, five female mosquitoes feed from a glass capillary filled with 10% sucrose, and the change in volume is measured to determine the amount of sugar solution ingested by the group over a two hour period (Figure 6A). To confirm that this assay measures sugar-feeding and not water-seeking, we carried out control experiments which indicated that fasted mosquitoes did not consume water alone from the capillary (data not shown). Non-fasted mosquitoes or those fasted for 12 hours consumed very little sugar in this assay (Figure 6B). After 24, 48, or 72 hours of fasting, there was an increase in sugar ingested (Figure 6B), but we observed high mortality after 72 hours of fasting. Based on these results, we analyzed sugar feeding in *npylr1^HR3/HR1^* mutants and associated genetic controls after 0 and 48 hours of fasting and found no difference between mutants and controls at either time point (Figure 6C).

To quantify sugar feeding by individual female mosquitoes more precisely, we designed the BUFFET assay. In this assay, fifty female mosquitoes were fasted for 48 hours and then allowed to feed on 10% sucrose+0.02% fluorescein presented in a 96-well micro-titer plate at the bottom of a mosquito cage (Figure 6D). After 2 hours, mosquitoes were individually homogenized to release the fluorescein from their gut, which was quantified by a fluorescent plate reader. Results from the BUFFET indicated that the amount of sugar ingested by individual *npylr1^8/4^* mutants did not differ from controls (Figure 6E).

To measure blood feeding, we used a membrane feeding assay with blood containing the fluorescent dye fluorescein. In this assay, fifteen female mosquitoes were given 15 minutes to feed from an artificial glass feeder containing warmed sheep blood mixed with 0.02% fluorescein (Figure 6F). Carbon dioxide (CO_2_), a general activator of mosquito behavior [31], [32], was added to the test environment to induce blood-feeding. Similar to the BUFFET, individual mosquitoes were then homogenized to release the fluorescein for quantification on a fluorescent plate reader. In these experiments, approximately 40% of both control and *npylr1* mutant mosquitoes successfully blood-fed (Figure 6G). We found no difference in the volume of blood ingested by *npylr1* mutants compared to controls (Figure 6H). The average volume of blood ingested was different between *npylr1^8/4^* and *npylr1^HR2/HR3^* experiments, but we believe this is an effect of using a different batch of sheep blood for each experiment or experimental variation from carrying out these experiments 3 months apart, rather than an effect of *npylr1* genotype on blood-meal size.

### Host-seeking behavior of *npylr1* mutants

**Figure 7:**
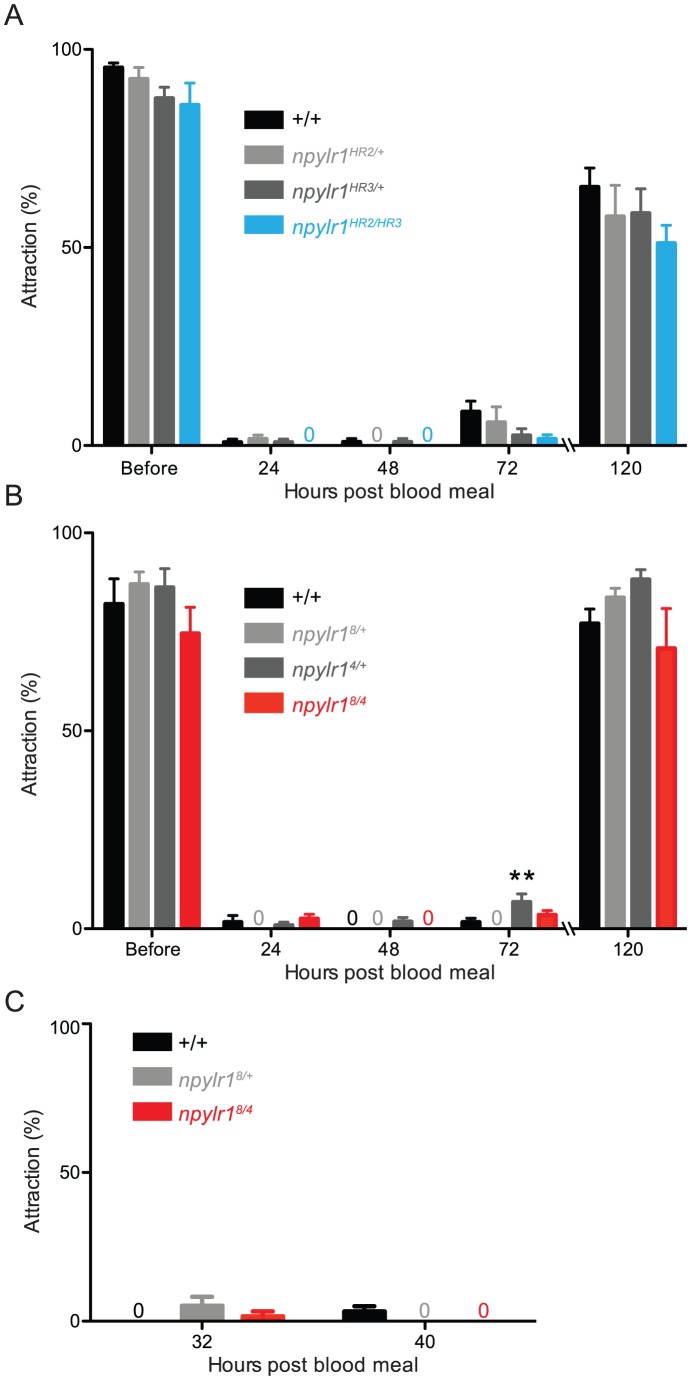
Normal host-seeking behavior of *npylr1* mutants throughout the gonotrophic cycle. Host-seeking behavior of *npylr1* (A) homologous recombination mutants and (B) non-homologous end-joining mutants with associated controls before and 24, 48, 72, and 120 hours after a blood meal. Egg-laying was observed after 72 hours and completed by 120 hours (n = 6–12, ∼20 females per trial). (C) Host-seeking behavior of *npylr1* homologous recombination mutants and associated controls at 32 and 40 hours after a blood meal (n = 3, ∼20 females per trial). All data are plotted as mean±SEM. 1-way ANOVA with Bonferroni correction for multiple comparisons within each time point.

Prior to a blood meal, female *npylr1* mutants displayed robust host-seeking behavior indicating no baseline defect in their response to host stimuli (Figure 7A and B). Following a blood meal, inhibition of host attraction was observed at 24 hours as expected from abdominal distension (Figure 7A and B). At 48 and 72 hours post-blood meal—time points suspected to be under the influence of Head Peptide-I [4], [10]—*npylr1^HR2/HR3^* and *npylr1^8/4^* displayed inhibition of host-seeking behavior at similar levels to controls (Figure 7A and B). Like wild-type mosquitoes, *npylr1* mutants recovered host-seeking behavior following egg deposition between 72 hours and 120 hours post-blood meal (Figure 7A and B). To ask whether *npylr1* mutants would show defects immediately following the relief of inhibition by abdominal distension at ∼24 hours post-blood meal, we increased the temporal resolution of these experiments to test at 32 and 40 hours post-blood meal. These experiments showed strong host-seeking suppression in all genotypes at both time points (Figure 7C).

## Discussion

The aim of this study was to identify receptors for Head Peptide-I as a means to clarify the mechanism of host-seeking behavior inhibition following a blood meal. We carried out a cell-based assay that identified peptide ligands for four of eight previously uncharacterized *Ae. aegypti* NPYLRs. These experiments identified NPYLR1 as the only receptor sensitive to Head Peptide-I and showed that this receptor was also very sensitive to sNPFs. We then showed that injection of either synthetic NPYLR1 ligand, Head Peptide-I or sNPF-3, into non-blood-fed female mosquitoes inhibited their normal high levels of host-seeking behavior. To ask if NPYLR1 was necessary for blood-feeding-induced host-seeking inhibition, five independent null mutant *npylr1* alleles were generated and tested for behavioral abnormalities. Our results showed that none of the *npylr1* mutants displayed abnormal behaviors in locomotion, egg-laying, sugar-feeding, blood-feeding, host-seeking, or inhibition of host-seeking after a blood meal.

**Figure 8:**
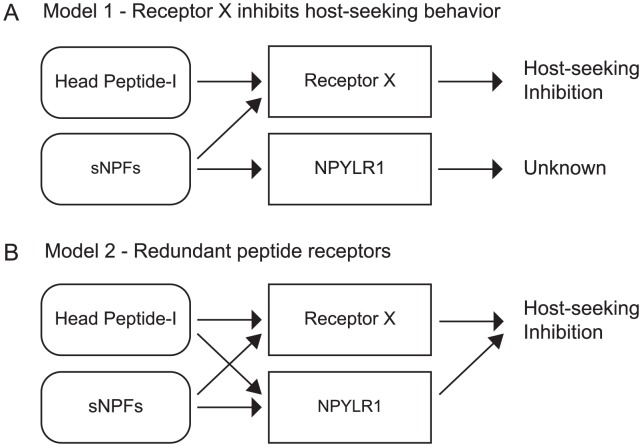
Models for the inhibition of host-seeking behavior in *Ae. aegypti*. (A) Model 1 illustrates the hypothesis that known peptides including Head Peptide-I and sNPFs signal through an unknown receptor (“Receptor X”) and that NPYLR1 is not involved in the inhibition of host-seeking behavior. (B) Model 2 illustrates the hypothesis that a combinatorial network of redundant mechanisms for behavioral inhibition may exist after blood-feeding.

Although our cell-based assay experiments suggested that NPYLR1 may contribute to Head Peptide-I signaling, *npylr1* mutants displayed no changes in the inhibition of host-seeking behavior. This result may indicate that the low sensitivity of NPYLR1 to Head Peptide-I found in the cell-based assay is not physiologically relevant. The primary function of NPYLR1 may be to respond to sNPFs, but the relationship of this receptor to the regulation of mosquito feeding behavior is still unclear. Alternatively, NPYLR1 may function redundantly with other receptors to modulate mosquito behavior. Based on our findings, we propose two models for the regulation of host-seeking behavior. In Model 1, an unknown receptor (“Receptor X”) is sensitive to Head Peptide-I and/or sNPFs and produces behavioral inhibition (Figure 8A). In Model 2, cross-talk between the NPYLR1 signaling pathway and putative additional redundant peptide receptors inhibits behavior (Figure 8B). In both cases, we believe an additional receptor must be involved in the inhibition of host-seeking behavior. This unknown receptor may be one of the four NPYLRs that failed to respond in our assay or another receptor that was not examined. The ease with which mosquito genes can now be knocked out with ZFNs [16] or TALENS [33], makes it feasible to generate mutants in all eight *Ae. aegypti npylr* genes to test these possibilities. Future validation of a redundant receptor may require behavioral analysis in an *npylr1* mutant background.

It is important to note several discrepancies between the *in vitro* cell-based assay and *in vivo* behavior experiments. First, the cell-based assay indicated that NPYLR1 was ten times more sensitive to sNPF-3 than Head Peptide-I. However, injection of either peptide at identical concentrations caused similar levels of host-seeking inhibition. The absence of stronger inhibition from sNPF-3 could be explained by increased stability of Head Peptide-I in the hemolymph due its post-translational modifications [9]. Second, in our cell-based assay, NPYLR1 showed similar sensitivity to Head Peptide-I and Head Peptide-III, which is behaviorally inactive and present at low concentrations in the hemolymph of blood-fed females [10]. Head Peptide-III differs from Head Peptide-I in lacking the post-translational modification of proline to hydroxyproline at position 4 [10]. Therefore, there is no clear connection between peptide-receptor activity in the cell-based assay and efficacy of these peptides as behavioral inhibitors *in vivo*. Additional factors such as peptide stability or access of the injected peptide to the cognate receptor must be at play in the mosquito.

Given our results and recent proteomic work published by others [22], the role of Head Peptide-I in *Ae. aegypti* remains unclear. Initial findings described Head Peptide-I as the most abundant RFamide in *Ae. aegypti* [22], but recent proteomic work failed to detect this peptide in the brain or midgut [22], tissues where it was previously localized [34]. Other investigators have shown that Head Peptide-I is synthesized in the male-accessory gland and transferred to the female during copulation [9], begging the question of whether Head Peptide-I is actually a neuropeptide rather than a male accessory gland factor. So the question remains, is Head Peptide-I involved in the inhibition of host-seeking behavior? Our experiments focused on NPYLR1 as a candidate Head Peptide-I receptor and therefore our results are unable to clarify a role for the peptide itself. The generation of a Head Peptide-I mutant for behavioral analysis and the re-examination of hemolymph content after a blood meal to identify additional candidate peptides may be fruitful pursuits for future studies.

While NPYLR1 was the only *Ae. aegypti* NPYLR found to be sensitive to Head Peptide-I in this study, it is much more sensitive to sNPFs. This finding is in accord with the phylogeny of NPYLR1, which is most closely related to known sNPF receptors in *D. melanogaster* and *An. gambiae*. Studies in *D. melanogaster* showed that sNPFs influence overall body size [19], [20] and modulate olfactory perception after fasting to influence food-search behavior [18]. We did not observe any obvious changes to body size in *npylr1* mutants. NPYLR8 is the likely orthologue of the *D. melanogaster* NPF receptor, which regulates food-search behavior and ingestion of noxious food in *D. melanogaster* larvae [35]. Lastly, we identified ligands for NPYLR5 and NPYLR7, but their role in insect behavior is unknown. Future genetic analysis of additional *Ae. aegypti npylr* mutants may clarify the biological function of each of these new receptors.

We are intrigued by the identification of a putative Head Peptide gene and functional validation of two NPYLR1 orthologues in the tick, *Ix. scapularis*. Ticks display intermittent feeding behaviors similar to *Ae. aegypti* but on the much longer time scale of months between successive blood meals [25], [26]. The conservation of Head Peptide-I signaling in ticks and mosquitoes suggest that this signaling pathway may also be present in other intermittent blood feeders such as lice and bed bugs. The increase in affordable and rapid genome sequencing of a large number of arthropod species brings the prospect of future evidence for a functional correlation between Head Peptide-I and intermittent blood feeding in arthropods.
